# Spontaneous Heterotopic Pregnancy with Unaffected Intrauterine Pregnancy: Systematic Review of Clinical Outcomes

**DOI:** 10.3390/medicina56120665

**Published:** 2020-11-30

**Authors:** Mihaela Oancea, Razvan Ciortea, Doru Diculescu, Alexandra-Andreea Poienar, Mihaela Grigore, Roxana-Adelina Lupean, Renata Nicula, Diana Chira, Stefan Strilciuc, Dan Mihu

**Affiliations:** 1 Department of Obstetrics and Gynaecology, ‘Iuliu Hatieganu’ University of Medicine and Pharmacy, 400006 Cluj-Napoca, Romania; mihaelaoancea321@yahoo.com (M.O.); r_ciortea@yahoo.com (R.C.); ddiculescu@yahoo.com (D.D.); roxanalupean92@gmail.com (R.-A.L.); renatanicula@yahoo.com (R.N.); dan.mihu@yahoo.com (D.M.); 2 Department of Obstetrics and Gynaecology, ‘Grigore T Popa’ University of Medicine and Pharmacy, 700115 Iasi, Romania; mihaela.grigore@edr.ro; 3 RoNeuro Institute for Neurological Research and Diagnostic, 400354 Cluj-Napoca, Romania; diana.chira@ssnn.ro; 4 Department of Neurosciences, ‘Iuliu Hatieganu’ University of Medicine and Pharmacy, 400006 Cluj-Napoca, Romania

**Keywords:** heterotopic, ectopic, pregnancy, diagnosis

## Abstract

*Background and objective*: Spontaneous heterotopic pregnancy (SHP) is a rare condition represented by the synchronous coexistence of an intrauterine and an ectopic pregnancy. It rarely occurs with natural conception and is usually a consequence of assisted reproductive techniques. Diagnosis of SHP can be a challenge for the clinician. The evolution of the intrauterine pregnancy is dependent on many factors, such as the location of the heterotopic pregnancy, gestational age at the time of diagnosis, the surgical procedure, the presence of other risk factors, early or delayed management. The aim of this systematic review of the literature was to extract existing evidence on spontaneous heterotopic pregnancy with otherwise unaffected intrauterine pregnancy. *Materials and Methods*: From a total of 1907 database entries identified in PubMed, EMBASE and Cochrane reviews, we selected 18 papers for narrative synthesis, for which we explored the diagnostic options, treatment, and outcome of these extremely rare epidemiologic occurrences. Manuscripts were assessed using the CARE guidelines for reporting case reports. *Results*: The main symptom was abdominal pain, and the preferred treatment approach was surgical, more precisely, using a laparoscopic approach. Most cases presented no risk factors, and the diagnosis was mostly made in the first semester. *Conclusions*: Normal follow-up and evolution of intrauterine pregnancy have been observed regardless of surgical approach (open or laparoscopic). Early diagnosis and treatment are advised, as they impact maternal and fetal outcomes. Evidence on this topic is scarce, predominantly comprised of case reports with variable degrees of adherence to dissemination guidelines. More studies on this topic are required to optimize care protocols for this type of pregnancy.

## 1. Introduction

Heterotopic pregnancy is the co-occurrence of ectopic pregnancy and intrauterine pregnancy. It is a pathological form of a dizygotic, biovular twin pregnancy [1] where one egg will nidate inside the uterine cavity and the other one will stop progression towards the uterus. The cause of this is an ovulatory abnormality or a difference in the migration speed of the two embryos, due to a delay in the capture of the fertilized egg by the fallopian tube [2]. It is a rare condition with an incidence of approximately 1 per 30,000 pregnancies [3] and can be potentially fatal [4,5].

The prevalence of heterotopic pregnancy has seen an increasing trend in the last decades, which may be attributed to the increased use of ovulation induction [6] and medically assisted pregnancies [7], rarely occurring with natural conception [8,9,10]. Additionally, patients who require assisted reproductive procedures often present with tubal pathology, which is one of the main causes of extrauterine pregnancy [11].

The risk factors for heterotopic pregnancy are very similar to those for ectopic pregnancy, including smoking, history of ectopic pregnancy, previous inflammatory pelvic disease, sexually transmitted infections (especially Chlamydia infections), surgery of the fallopian tubes, abdominal surgery, endometriosis, infertility treatments, and some forms of contraception [7].

Diagnosis is often times extremely difficult due to the intrauterine pregnancy masking the ectopic one [12]. Lower levels of the β-subunit of human chorionic gonadotropin (HCG) are usually an indicator of ectopic pregnancy [12]. Endovaginally ultrasonography allows correct diagnosis in 88.9% of cases by revealing the actual intrauterine and extrauterine pregnancies [2]. In case of uncertainty after performing the ultrasound, an exploratory laparoscopic intervention may be performed in order to facilitate diagnosis and subsequent steps in clinical management [13].

The definitive result is provided by the pathology department which may describe chorionic villi in the wall of the tube, confirming the presence of an ectopic gestation. It may also describe inflammation and distortion of plicae, and modifications consistent with chronic salpingitis [14].

The treatment of heterotopic pregnancy consists of surgical intervention in order to remove the extrauterine pregnancy. A laparoscopic approach is usually desirable in the absence of contraindications. In this case, the intrauterine pregnancy is preserved and may advance with normal surveillance and with no additional complications [15]. The overall prognosis for spontaneous heterotopic pregnancy (SHP) is similar to extrauterine pregnancy, depending on the management of the extrauterine pregnancy. Fetal prognosis remains reserved and mostly uncertain even after treatment, as approximately 35% of cases eventually develop into miscarriages. Functional prognosis is influenced by the approach used in treatment and the biological heterogeneity of patients [2].

To date, there is no systematic review published that synthesized SHP. In this qualitative synthesis, we aim to identify the best options for the diagnosis, treatment, and outcome of this extremely rare condition.

## 2. Materials and Methods

We performed a multi-database review of the literature on the topic, focusing on reports of live births after SHP which describe the clinical presentation, diagnostic and therapeutic procedures. The rationale for selecting live births as a strict inclusion criterion is the issue of uncertain causality between the interaction of various pathophysiological mechanisms that lead to SHP and associated treatments with clinical outcome.

The protocol of the systematic review and meta-analysis was written in compliance with the Preferred Reporting Items for Systematic Reviews and Meta-Analyses (PRISMA) statement [16], and with requirements of the international prospective register of systematic reviews (PROSPERO). Due to substantial delays in protocol publication acknowledged by the National Institute of Health Research (NIHR) in the United Kingdom, the registration number is not available at this time.

The PICOS criteria used for this review were the inclusion of original case reports or case series (study design) of females presenting with spontaneous heterotopic pregnancy after natural conception (population) who were diagnosed, treated and followed up in specialized secondary and tertiary care (intervention). The cases of patients who underwent assisted reproductive procedures were excluded.

We performed a systematic search using PubMed (MEDLINE), EMBASE, and Cochrane review databases between 21st September 2019 and 12th October 2020, with no restrictions for date of publication or study design. Due to the scarcity of resources for screening, interpreting, and reporting data, we only included articles published in English with available full-text manuscripts.

The search strategy for PubMed was converted to an exact corresponding match for EMBASE and Cochrane reviews: “pregnancy, heterotopic” [MeSH Terms] OR (“pregnancy” [All Fields] AND “heterotopic” [All Fields]) OR “heterotopic pregnancy” [All Fields] OR (“heterotopic” [All Fields] AND “pregnancy” [All Fields]).

After a formal search, all query entries were pooled into a spreadsheet for duplicate removal based on the title, author(s) and journal names. Two operators individually screened for relevance all search results (abstracts, and subsequently full-text manuscripts) based on the PICOS criteria, using automated open-source software.

Data from the articles selected for inclusion in the qualitative synthesis of the systematic review were extracted independently by study operators in a tailored spreadsheet containing the following variables: author name(s), journal name, date of publication, patient demographics (age, risk factors), clinical presentation (onset symptoms, gestational age, treatment type, outcome, complications). The existing information was simplified and recoded using dichotomization (where applicable) and allocation to nominal categories.

**Figure 1:**
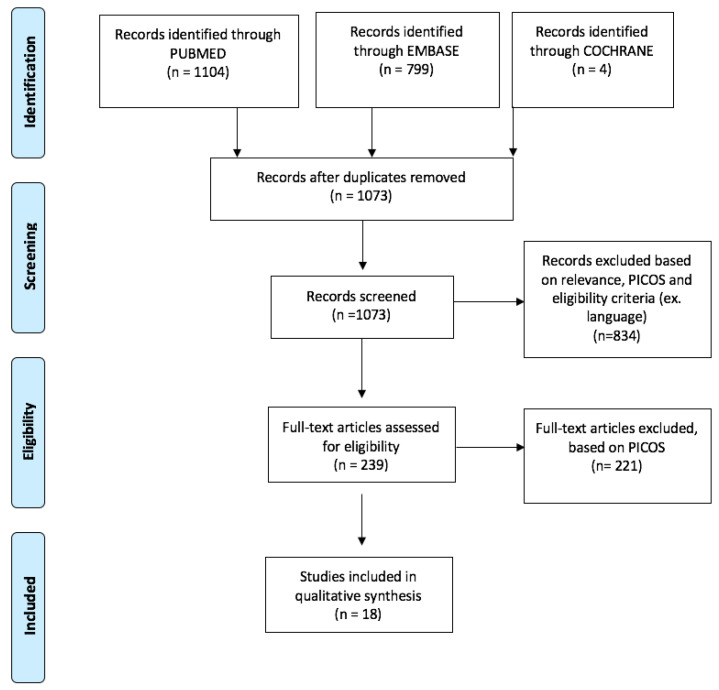
PRISMA flowchart of systematic review article selection process.

A total of 1907 articles were retrieved using the search strategy (PubMed = 1104, EMBASE = 799, Cochrane reviews = 4). After duplicate removal, screening, full-text assessment based on PICOS 18 articles were finally included in the qualitative synthesis. The flowchart of the selection process is available in Figure 1.

**Figure 2:**
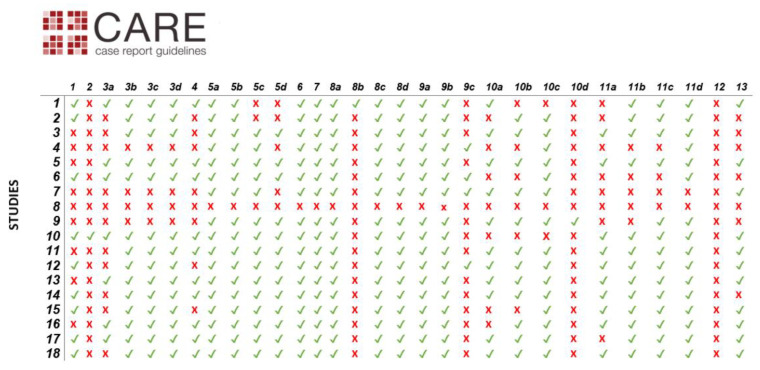
Assessment of selected articles using the CARE guidelines. Diagnostic challenges (**8b**), changes in therapeutic interventions (**9c**), adverse and unanticipated events (**10d**), and patient perspectives (**12**) are grossly underreported in the identified case reports. Legend: (**1**) Title—The diagnosis or intervention of primary focus followed by the words “case report”; (**2**) Key Words—2 to 5 key words that identify diagnoses or interventions in this case report (including “case report”); (**3a**) Introduction—What is unique about this case and what does it add to the scientific literature; (3b) The patient’s main concerns and important clinical findings; (**3c**) The primary diagnoses, interventions, and outcomes; (**3d**) Conclusion—“Take-away” lessons from this case report? (**4**) Introduction—Briefly summarizes why this case is unique and may include medical literature references; (**5a**) De-identified patient specific information; (**5b**) Primary concerns and symptoms of the patient; (**5c**) Medical, family, and psychosocial history including relevant genetic information; (**5d**) Relevant past interventions and their outcomes; (**6**) Describe significant physical examination (PE) and important clinical findings; (**7**) Historical and current information from this episode of care organized as a timeline (figure or table); (**8a**) Diagnostic methods (PE, laboratory testing, imaging, surveys); (**8b**) Diagnostic challenges; (**8c**) Diagnosis (including other diagnoses considered); (**8d**) Prognostic characteristics when applicable; (**9a**) Types of therapeutic intervention (pharmacologic, surgical, preventive); (**9b**) Administration of therapeutic intervention (dosage, strength, duration); (**9c**) Changes in therapeutic interventions with explanations; (**10a**) Clinician- and patient-assessed outcomes if available; (**10b**) Important follow-up diagnostic and other test results; (**10c**) Intervention adherence and tolerability; (**10d**) Adverse and unanticipated events; (**11a**) Strengths and limitations in this case; (**11b**) Discussion of the relevant medical literature; (**11c**) The rationale for the conclusions; (**11d**) The primary “take-away” lessons from this case report; (**12**) The patient should share their perspective on the treatment(s) they received; (**13**) The patient should give informed consent.

Assessment of risk of bias was performed at study level based on the CARE guidelines [17], using parts of the checklist as an eligibility benchmark for qualitative synthesis: patient information, clinical findings, timeline, diagnostic and clinical assessment, therapeutic intervention, follow-up and outcomes, discussion. The absence of the patient’s perspective was not enforced as a reason for exclusion. Informed consent was presumed to be available, even if not explicitly mentioned in all articles. Assessment of the selected articles using the CARE guidelines is available in Figure 2.

## 3. Results

A detailed account of the study characteristics, including variables collected as part of the synthesis of information, is included in Table 1. Maternal age ranged from 21 to 37 years. Diagnosis was mainly established in the first trimester, the mean gestational age being 8 weeks. The most frequent complication associated with spontaneous heterotopic pregnancy was tubal rupture (13/18 articles). Diagnosis was established between 6–20 weeks of gestation (median = 8 weeks). Only three reports mentioned the race and ethnicity of the women (two Hispanic, one Southeast Asian).

The majority of heterotopic pregnancies presented with abdominal pain as the main symptom [18,19], as shown by our review. Other presentations include left lower quadrant pain or right lower quadrant pain, vaginal spotting or bleeding [20,21,22].

Extrauterine pregnancies were mostly located in the fallopian tubes. The preferred treatment approach was surgical, and the procedure was mostly done in an open manner, using laparoscopy (*n* = 8). Seven articles reported the use of laparotomy, and there was only one aspiration, one hysteroscopy and one salpingectomy. Most women had an uneventful antenatal course, delivering healthy babies. All patients had a successful recovery, with no deaths occurring in the reported cases. The majority of the articles studied by us reported the presence of hemoperitoneum.

Some cases do not present any risk factors [23,24]. The majority of the studies included in our review presented cases with no risk factors. The identified articles reported risk factors such as a history of previous abdominal surgery, intrauterine interventions or pelvic infections. Another risk factor cited in the literature is smoking; therefore, special attention must be given to smoking patients [19,25].

Diagnosis was usually made in the first trimester. We found only one article describing a diagnosis of heterotopic pregnancy in the second trimester, at 20 weeks of gestation [19]. Because ectopic pregnancy is usually located in the tubal area, diagnosis is made in the first trimester due to the abdominal pain secondary to the dilation of the fallopian tube and its rupture, thus resulting in hemoperitoneum.

The main issues from the perspective of risk of bias in reported cases, as assessed by the CARE guidelines, are incomplete patient information, inaccurate reporting of timelines (e.g., diagnosis in the first trimester), incomplete maternal follow-up, and unspecified use of informed consent (Figure 2).

**Table 1:** Study Characteristics. Legend: LLQ—left lower quadrant; RLQ—right lower quadrant; D&C—dilation and curettage; IUP—intrauterine pregnancy.

Article	Authors	Year	Risk Factors	Onset Symptoms	Gestational Age at Diagnosis	Type of Intervention	Complication	IUP Outcome	Location of Ectopic Pregnancy
1	Lialios et al. [26]	2008	-	Abdominal pain	First trimester	laparoscopy	Tubal rupture	vaginal delivery	interstitial
2	Tandon et al. [27]	2009	-	Abdominal pain	8 w	laparotomy	Tubal rupture	vaginal delivery	tubal
3	Phupong et al. [25]	2010	+	Abdominal pain	7 w	laparoscopy	Tubal rupture	cesarean delivery	tubal
4	Uysal et al. [28]	2013	+	Vaginal bleeding	6 w	aspiration	-	vaginal delivery	cervical
5	Ikechukwu et al. [23]	2013	+	Abdominal pain	10 w	laparotomy	Tubal rupture	vaginal delivery	ampullary
6	Chadee et al. [18]	2016	+	Pain of LLQ	8 w	laparotomy	Tubal rupture	D&C on demand	ampullary
7	Okunowo et al. [19]	2016	-	Abdominal pain	20 w	laparotomy	Tubal rupture	unaffected IUP	abdominal
8	Bataille et al. [29]	2017	-	Abdominal pain	6–14 w	laparotomy	Tubal rupture	vaginal delivery	tubal
9	Xie et al. [30]	2018	-	Abdominal pain	12 w + 2 d	laparoscopy	Tubal rupture	vaginal delivery	tubal
10	Ciebiera et al. [4]	2018	-	Abdominal pain	13 w	laparotomy	Tubal rupture	vaginal delivery	tubal
11	Guimarães et al. [31]	2019	-	Abdominal pain	8 w	laparotomy	Tubal rupture	cesarean delivery	tubal
12	Ramalho et al. [32]	2019	-	Pain of RLQ	6 w	laparoscopy	-	vaginal delivery	ovarian
13	Stanic et al. [33]	2019	-	Vaginal bleeding/Abdominal pain	6/7 w/12 w	laparoscopy	Tubal rupture	unaffected IUP	tubal
14	Cerniauskaite et al. [34]	2020	-	Abdominal pain	7 w	laparoscopy	-	vaginal delivery	tubal
15	Holley et al. [35]	2020	-	Abdominal pain	8 w	salpingectomy	-	unaffected IUP	cervical
16	Diakosavvas et al. [36]	2020	-	Abdominal pain	5 w	laparoscopy	Tubal rupture	vaginal delivery	tubal
17	Rubattu et al. [37]	2020	+	Vaginal bleeding	3 w	hysteroscopy	-	cesarean delivery	cervical
18	Aziz et al. [38]	2020	+	Abdominal pain	7 w	laparoscopy	Tubal rupture	spontaneous abortion	tubal

w = weeks; d = days; - = absent.

## 4. Discussion

This systematic review extracted available literature information regarding the diagnosis, treatment options and outcome of SHP after natural conception. Timely diagnosis can be difficult in the absence of specific symptoms.

The majority of patients presented with pelvic pain, accompanied by vaginal bleeding and amenorrhea [4,10]. This triad seems to be encountered by all patients described in the case reports we have identified. However, since these symptoms may be present in normal intrauterine pregnancies, thus early diagnosis can be mistaken for a much less serious condition [4,10]. Medical staff must not underestimate pregnant woman presenting to the emergency department with abdominal pain. It is recommended that intrauterine pregnancies are carefully investigated along with the adnexa and the abdominal cavity. The most common differential diagnoses of SHP are miscarriage, ectopic pregnancy, intrauterine pregnancy with hemorrhagic corpus luteum, and adnexal torsion. Non-gynecological causes, such as appendicitis, cholecystitis, bowel obstruction or pancreatitis, should also be excluded [32].

Ultrasonography is a valuable imaging tool in the challenging process of diagnosing spontaneous heterotopic pregnancy [9]. Since there are no specific investigations available to screen for SHP, clinicians must rely on clinical signs in conjunction with exhaustive ultrasound examination of the uterus and adnexa [4,9,10], or even resort to exploratory laparoscopy or laparotomy in cases where the ultrasonographic findings are unclear [32]. The presence of an intrauterine pregnancy does not exclude the presence of synchronous ectopic pregnancy. Careful examination of patients with normal intrauterine pregnancies who present the triad of amenorrhea, vaginal bleeding and pelvic pain is therefore mandatory [10].

In the majority of cases, diagnosis of SHP is made late, when rupture occurs, and patients present with hemoperitoneum. Early management is essential in order to avoid severe maternal complications. SHP has a higher incidence in patients with a history of infertility, following assisted reproductive techniques [5] and represents the reason why many fertility clinics prefer single-embryo transfers and rigorous ultrasonographical post-implantation follow-up [32]. However, our review highlighted the possibility of encountering this pathology as a consequence of natural conception. Although the presence of extrauterine pregnancy is usually associated with risk factors, we found a number of case reports that presented patients with heterotopic pregnancies in the absence of known risk factors [4,19,26,27,29,33,34,36]. Five articles mentioned the existence of risk factors for the patients, such as a history of pelvic inflammatory disease, history of repeated miscarriages and intrauterine interventions, history of abdominal surgery [18,23,30,38,39], thus emphasizing the importance of identifying patients with risk factors and performing differential diagnosis on individuals with clinical signs of SHP.

Race could not be assessed as a potential risk factor for SHP, since very scarce information is present in the literature. The choice between classic or laparoscopic approaches was reportedly based on the experience of surgeons, location of pregnancy, patient status and preference. Based on the identified evidence, there is no overarching pattern to suggest superiority of a particular surgical approach for SHP. Despite division across surgical techniques, minimal intraoperative manipulation of the uterus is advised in order to prevent ruptures and damage to the intrauterine pregnancy [40]. Emergency surgical treatment is advised in the presence of hemoperitoneum [4,18,19,21,23,26,27,28,31,33,36,38,40].

Medical treatment is described in the literature, such as ultrasound-guided injections of saline solutions in the ectopic sack [31] but none of the articles included in our review described using this technique.

The outcome of the intrauterine pregnancy depends on many factors, such as the maternal status at the moment of admission, the location of the extrauterine pregnancy. Hypovolemic shock of the mother can lead to a poor prognosis of the intrauterine pregnancy. Our review revealed improved outcomes for intrauterine pregnancies when the extrauterine ones were located in the fallopian tubes, as compared to the interstitial ectopic sack [26]. Almost all articles included in our study reported an unaffected intrauterine pregnancy, with the exception of one article that reported a spontaneous abortion [38]. However, the literature describes an unaffected intrauterine pregnancy in two thirds of cases and spontaneous abortion in one third [32].

## 5. Conclusions

In the case reports identified by our systematic search, successful follow-up and evolution of intrauterine pregnancy have been observed regardless of surgical approach (open or laparoscopic) after SHP. Early diagnosis and treatment are advised, as they impact maternal and fetal outcomes. Evidence on this topic is scarce, predominantly comprised of case reports with variable degrees of adherence to dissemination guidelines. An additional concern when synthesizing information from multiple case reports is publication bias, which should be considered when interpreting the information present in this manuscript, as the tendency is to select cases with successful outcomes. More studies on this topic are required to inform clinical guidelines and to optimize care protocols for the increasing occurrence of SHP.
